# Accuracy of Various Forms of Contrast-Enhanced MRI for Diagnosing Hepatocellular Carcinoma: A Systematic Review and Meta-Analysis

**DOI:** 10.3389/fonc.2021.680691

**Published:** 2021-12-07

**Authors:** Chun Zhao, Hongyan Dai, Juwei Shao, Qian He, Wei Su, Peng Wang, Qiuyue Tang, Junren Zeng, Song Xu, Juanjuan Zhao, Shutian Xiang

**Affiliations:** Department of Radiology, Affiliated Hospital of Yunnan University, Kunming, China

**Keywords:** hepatocellular carcinoma, magnetic resonance imaging, meta-analysis, validation studies, gadoxetic acid

## Abstract

**Background:**

Contrast-enhanced MRI can be used to identify patients with hepatocellular carcinoma (HCC). However, studies around the world have found differing diagnostic accuracies for the technique. Hence, we designed this meta-analysis to assess the accuracy of contrast-enhanced MRI for HCC diagnosis.

**Methods:**

We conducted a systematic search for all studies reporting the diagnostic accuracy of contrast-enhanced MRI for HCC in the databases of MEDLINE, EMBASE, Cochrane Library, Web of Science, SCOPUS, ScienceDirect, and Google Scholar from inception until January 2021. We used the “Midas” package from the STATA software to perform the meta-analysis.

**Results:**

Our study was based on 21 publications with 5,361 patients. The pooled HCC diagnosis sensitivity and specificity were 75% (95% CI, 70%–80%) and 90% (95% CI, 88%–92%), respectively, for gadoxetic acid-enhanced MRI; and they were 70% (95% CI, 57%–81%) and 94% (95% CI, 85%–97%), respectively, for MRI with extracellular contrast agents (ECA-MRI). We found significant heterogeneity with a significant chi-square test and an *I* ^2^ statistic >75%. We also found significant publication bias as per Deeks’ test results and funnel plot.

**Conclusion:**

We found that both types of contrast-enhanced MRI are accurate diagnostic and surveillance tools for HCC and offer high sensitivity and specificity. Further studies on different ethnic populations are required to strengthen our findings.

## Introduction

Several academic guidelines for diagnosis of non-invasive hepatocellular carcinoma (HCC) exist (1) including those by the “American Association for the Study of Liver Diseases (AASLD) (2, 3)”, the “Asian Pacific Association for the Study of the Liver (APASL) (4)”, the “European Association for the Study of the Liver (EASL) (5)”, the “Korean Liver Cancer Study Group-National Cancer Center (KLCSG-NCC) (6)”, and the “Liver Imaging Reporting and Data System (LIRADS) (7, 8)”. Updates to the AASLD in 2017 and 2018 added the LIRADS to the guidelines (7, 8). LIRADS is a diagnostic algorithm that reflects local variations in liver cancer incidence, resources, and diagnostic and therapeutic approaches (9). Thus, health practitioners in different locations need to consider the costs and benefits for each guideline and to choose the best-suited diagnostic criteria for their patients.

Western guidelines, such as LIRADS (7, 8) and EASL (5), were initially based on radiological signs revealed by extracellular contrast agents (ECAs). The subsequent definition of the portal venous washout appearance of HCCs under gadoxetic acid has helped prevent false-positive diagnoses. However, the Eastern guidelines (KLCSG-NCC and APASL) allow for the alternative finding of hypointensity in the transitional or hepatobiliary phases under gadoxetic acid-enhanced MRI (4, 5). All the guidelines recommend using more than one radiological imaging modality for difficult-to-diagnose cases. The diagnostic performances of the latest guideline versions share similar accuracies (10).

Early and precise diagnoses allow HCC detection when medical and interventional therapies have the greatest positive impact. Correct staging of the disease by clinicians and radiologists is important for the choice of treatment, as demonstrated in recent publications (11–13). Moreover, staging is important in both primary and recurrent HCCs, as published (14). Hence, our updated review should allow clinicians around the world to choose the most effective diagnostic tool available for their patients without having to recur to different methods. For example, a specific contrast-enhanced MRI may provide accurate diagnoses on its own) (15), and it may be the most cost-effective diagnostic approach for HCC (16). Hence, we systematically searched the literature for all studies reporting the accuracies of ECA-enhanced and gadoxetic acid-enhanced MRIs based on the latest guidelines, and we pooled the available evidence into a meta-analysis to establish the accuracy of contrast-enhanced MRIs for HCC.

## Materials and Methods

### Eligibility Criteria

#### Types of Studies

We included studies examining diagnostic accuracy of contrast-enhanced MRI methods for making specific HCC diagnoses (irrespective of study design). We excluded studies lacking the data needed to obtain pooled estimates of sensitivity and specificity, unpublished studies, and gray literature from our review.

#### Types of Participants

We included studies conducted among patients with a suspected HCC mass or patients with signs and symptoms of HCC.

#### Index Test

Eligible studies used contrast-enhanced MRI and either ECA or gadoxetic acid as a contrast agent according to one of the following criteria for HCC diagnosis: AASLD, APASL, EASL, KLCSG-NCC, or LIRADS.

#### Reference Standards

Eligible studies compared contrast-enhanced MRI with histopathological examination or biopsy as the reference standard.

### Outcome Measures

Our outcome measures included sensitivity, specificity, diagnostic odds ratio (DOR), positive likelihood ratio (+LR), and negative likelihood ratio (−LR).

### Search Strategy

We used a detailed and predefined literature search strategy in MEDLINE, EMBASE, Cochrane Library, Web of Science, SCOPUS, ScienceDirect, and Google Scholar databases. We used the following MeSH terms: “Magnetic Resonance Imaging”, “Contrast MRI”, “Gadoxetic Acid”, “Extracellular MRI Contrast Agent”, “Hepatocellular Carcinoma”, “Diagnostic Accuracy Studies”, “Liver Tumours”, “Tumours of the Liver”, “Validation Studies”, and “Liver Malignancy”. We searched each database from inception until January 2021 without any language restriction. We also cross-checked the bibliographies of retrieved studies and hand-searched them for any articles satisfying the inclusion criteria.

### Study Selection

Two authors performed the primary literature search screening (i.e., title, keywords, and abstract screening). They retrieved the full text of all the promising articles. Then, they screened the retrieved full texts and assessed their eligibility against the predefined criteria. Disagreements about the inclusion of articles were resolved with the help of a third author. The fourth investigator helped to ensure the quality of the entire review process.

### Data Extraction

The primary author was responsible for extracting the data from the final set of selected studies. We developed a predefined data extraction template to obtain the following set of variables: authors, year of publication, study period, study design, setting, country/region, total sample size, reference standard, average age, quality-related parameters, sensitivity, and specificity. In addition, the primary investigator transferred the extracted data into the STATA version 14 software. A second investigator double-checked the data entry for correctness by comparing the data in our review and those in the retrieved articles.

### Risk of Bias Assessment

Two authors independently used the “*Quality assessment of diagnostic accuracy studies-2 (QUADAS-2)*” tool to assess the bias risk on the following domains: patient selection, index and reference test execution and interpretation, and outcome assessment flow and timing (17). We graded the studies as having high or low risk of bias based on the findings from these domains.

### Statistical Analysis

We conducted a bivariate meta-analysis to obtain the pooled sensitivity and specificity of contrast-enhanced MRI for HCC diagnosis. We performed the analysis based on the type of contrast agent and the guidelines used to obtain separate pooled estimates. We also calculated +LR, −LR, and the DOR to determine the utility of contrast-enhanced MRI. We graphically represented the pooled specificity and sensitivity with a Forest plot, the +LR and −LR with an LR scattergram, and the pretest and posttest probability of HCC diagnosis with a *t* and Fagan’s plot. Additionally, we plotted the “*Summary receiver operator characteristic curve (sROC)*” to assess the HCC diagnostic accuracy.

We evaluated the heterogeneity between studies using three methods: a chi-square test to determine the presence of heterogeneity, the *I* ^2^ statistic to quantify the heterogeneity, and a bivariate boxplot to graphically represent the heterogeneity. We found a high level of heterogeneity and performed a meta-regression to identify its source. The covariates adjusted during this meta-regression included study design, country, sample size, contrast agent, guidelines, average age, and quality-related factors. We used Deeks’ test and a graphical representation with a funnel plot to assess publication bias. We also performed a sensitivity analysis to detect any significant influence of a single study effect on the pooled estimate. We used the STATA software “Midas” command package to perform all analyses.

## Results

### Study Selection

**Figure 1:**
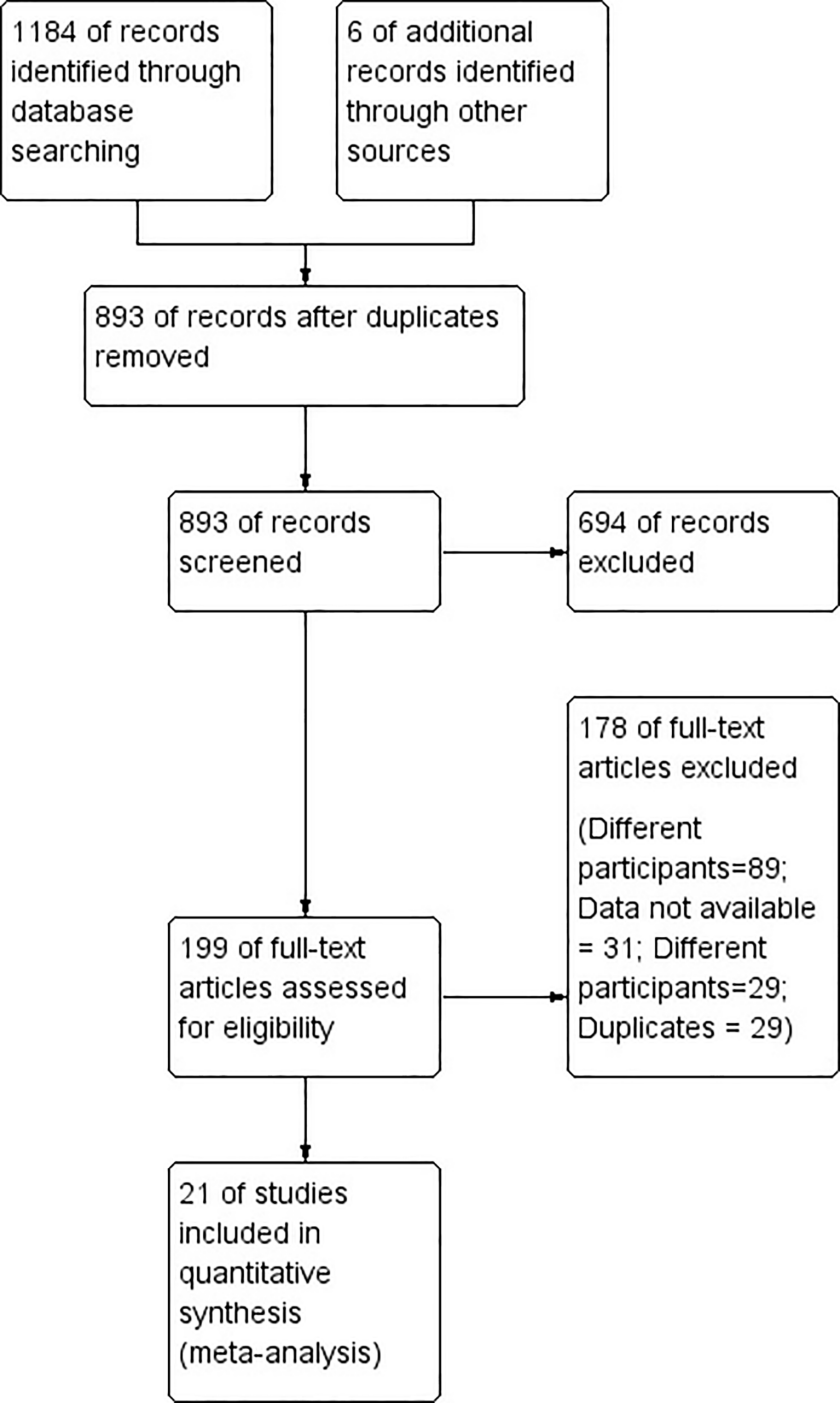
Search strategy.

**Figure 1** shows the entire study selection process in the form of a Preferred Reporting Items for Systematic Reviews and Meta-Analyses (PRISMA) flowchart. During our primary screening, we retrieved 266 full-text studies that totaled 199 after the removal of duplicates. Additionally, we identified six more articles from the manual search of the bibliographies. We ended up including 21 studies with 5,361 participants satisfying the inclusion criteria after our secondary screening (**Figure 1**) (10, 18–37).

### Study Characteristics

Most studies (17 out of 21) were retrospective, and four were prospective in nature. Most studies (17 out of 21) were conducted in Asian countries such as Korea (13) and China (4). The mean ages of the patients in the studies ranged from 49.5 to 66 years. Sample sizes ranged from 52 to 792 individuals. We found 18 studies that used gadoxetic acid contrast-enhanced MRI and eight studies that used ECA-MRI. Five studies diagnosed HCC based on APASL, six based on EASL guidelines, seven based on KLCSG-NCC, and 19 based on LIRADS guidelines (**Table 1**).

**Table 1:** Characteristics of the included studies (n = 21).

Study no.	First author and year	Country	Study design	Sample size	Study participants	Type of contrast agent	Type of current guidelines	Reference standard	Mean age (in years)
1	Ayuso et al., 2019 (18)	Spain	Prospective	52	Asymptomatic patients with new Child‐Pugh A–B cirrhotic US‐detected solitary nodules	Gadoxetic acid-enhanced MRI	AASLD	Biopsy	66
2	Byun et al., 2020 (19)	Korea	Retrospective	493	Patients with a) focal hepatic solid nodules detected on MRI, b) nodule size 1.0–3.0 cm, c) ≤5 nodules present, d) nodules benign on MRI	Gadoxetic acid-enhanced MRI	AASLD, EASL, KLCSG-NCC, LIRADS	Histopathology	59.7
3	Cha et al., 2020 (20)	Korea	Prospective	147	Patients with chronic hepatitis B or liver cirrhosis without prior treatment history of HCC	Gadoxetic acid-enhanced MRI, ECA-MRI	LIRADS	Histopathology	55
4	Chen et al., 2020 (21)	China	Retrospective	174	Adults (≥18 years) cirrhosis or chronic hepatitis B virus infection even in the absence of cirrhosis	ECA-MRI	LIRADS	Histopathology	49.5
5	Hwang et al., 2021 (10)	Korea	Retrospective	241	Patients with risk factors for HCC (chronic hepatitis B or liver cirrhosis of any etiology)	Gadoxetic acid-enhanced MRI	AASLD, EASL, KLCSG-NCC, LIRADS	Histopathology	58
6	Jeon et al., 2020 (12)	Korea	Retrospective	154	Patients with gadoxetic acid-enhanced liver MR imaging within 3 months before liver transplantation	Gadoxetic acid-enhanced MRI	AASLD, EASL, KLCSG-NCC, LIRADS	Histopathology	54.1
7	Jiang et al., 2019 (23)	Korea	Prospective	229	Adult patients with hepatitis B virus infection and/or cirrhosis	Gadoxetic acid-enhanced MRI	EASL and LIRADS	Histopathology	51.2
8	Kang et al., 2020 (24)	Korea	Prospective	103	Age ≥18 years; at risk for HCC with liver cirrhosis or chronic hepatitis B viral infection, and at least one treatment-naïve solid hepatic observation	Gadoxetic acid-enhanced MRI	EASL and KLCSG-NCC	Histopathology	63.1
9	Khatri et al., 2020 (35)	United States	Retrospective	86	Adult patients with medical record history of cirrhosis; unequivocal cirrhotic liver morphology on diagnostic imaging	ECA-MRI	LIRADS	Histopathology	57.7
10	Kierans et al., 2019 (26)	United States	Retrospective	92	Patients >18 years with a clinical diagnosis of cirrhosis, chronic hepatitis B or C infection	ECA-MRI and gadoxetic acid-enhanced MRI	LIRADS	Histopathology	57
11	Kierans et al., 2019 (27)	United States	Retrospective	159	Patients with a clinical diagnosis of cirrhosis or chronic hepatitis B with or without cirrhosis	ECA-MRI and gadoxetic acid-enhanced MRI	LIRADS	Histopathology	56.5
12	Kim et al., 2019 (28)	Korea	Retrospective	220	Patients with liver cirrhosis and histopathologically diagnosed mass-forming hepatic malignancies	Gadoxetic acid-enhanced MRI	LIRADS	Histopathology	58
13	Kim et al., 2020 (29)	Korea	Retrospective	165	Patients at risk of HCC with pathologically confirmed PLC	Gadoxetic acid-enhanced MRI	LIRADS	Histopathology	58
14	Ko et al., 2019 (30)	Korea	Retrospective	137	Patients at high risk of developing HCCs according to the AASLD guidelines	Gadoxetic acid-enhanced MRI	LIRADS	Histopathology	57.9
15	Lee et al., 2019 (31)	Korea	Retrospective	422	Patients >18 years; at risk for HCC according to LIRADS v2017 (cirrhosis/chronic hepatitis B)	Gadoxetic acid-enhanced MRI	LIRADS	Histopathology	59
16	Lee et al., 2019 (32)	Korea	Retrospective	218	Patients >18 years with at least one and up to five hepatic lesions (each ≥1 cm) on MRI	ECA-MRI and gadoxetic acid-enhanced MRI	EASL and LIRADS	Histopathology	57.1
17	Lee et al., 2020 (33)	Korea	Retrospective	266	Patients >18 years; at high risk for HCC with cirrhosis or chronic hepatitis B	ECA-MRI and gadoxetic acid-enhanced MRI	EASL and LIRADS	Histopathology	57.4
18	Park et al., 2020 (34)	Korea	Retrospective	792	Patients who underwent liver surgery within 6 months from the date of the MRI exam	Gadoxetic acid-enhanced MRI	LIRADS	Histopathology	56.2
19	Park et al., 2021 (35)	Korea	Retrospective	447	All patients with focal solid nodules observed on MRI, nodules 1–3 cm in size, and ≤3 nodules	Gadoxetic acid-enhanced MRI	AASLD, EASL, KLCSG-NCC, LIRADS	Histopathology	56.4
20	Ren et al., 2019 (36)	China	Retrospective	217	Patients with hepatic lesions who had HCC risk factors and underwent diagnostic MRI with ECA	ECA-MRI	LIRADS	Histopathology	NA
21	Zhang et al., 2019 (37)	China	Retrospective	245	Patients with high risk of HCC [hepatitis B, hepatitis C infection, or cirrhosis]	Gadoxetic acid-enhanced MRI	LIRADS	Histopathology	50.3

US, ultrasound; AASLD, American Association for the Study of Liver Diseases; EASL, European Association for the Study of the Liver; KLCSG-NCC, Korean Liver Cancer Study Group-National Cancer Center; LIRADS, Liver Imaging Reporting and Data System; HCC, hepatocellular carcinoma; ECA, extracellular contrast agent; PLC, primary liver cancer.

#### Risk of Bias Assessment

**Figure 2:**
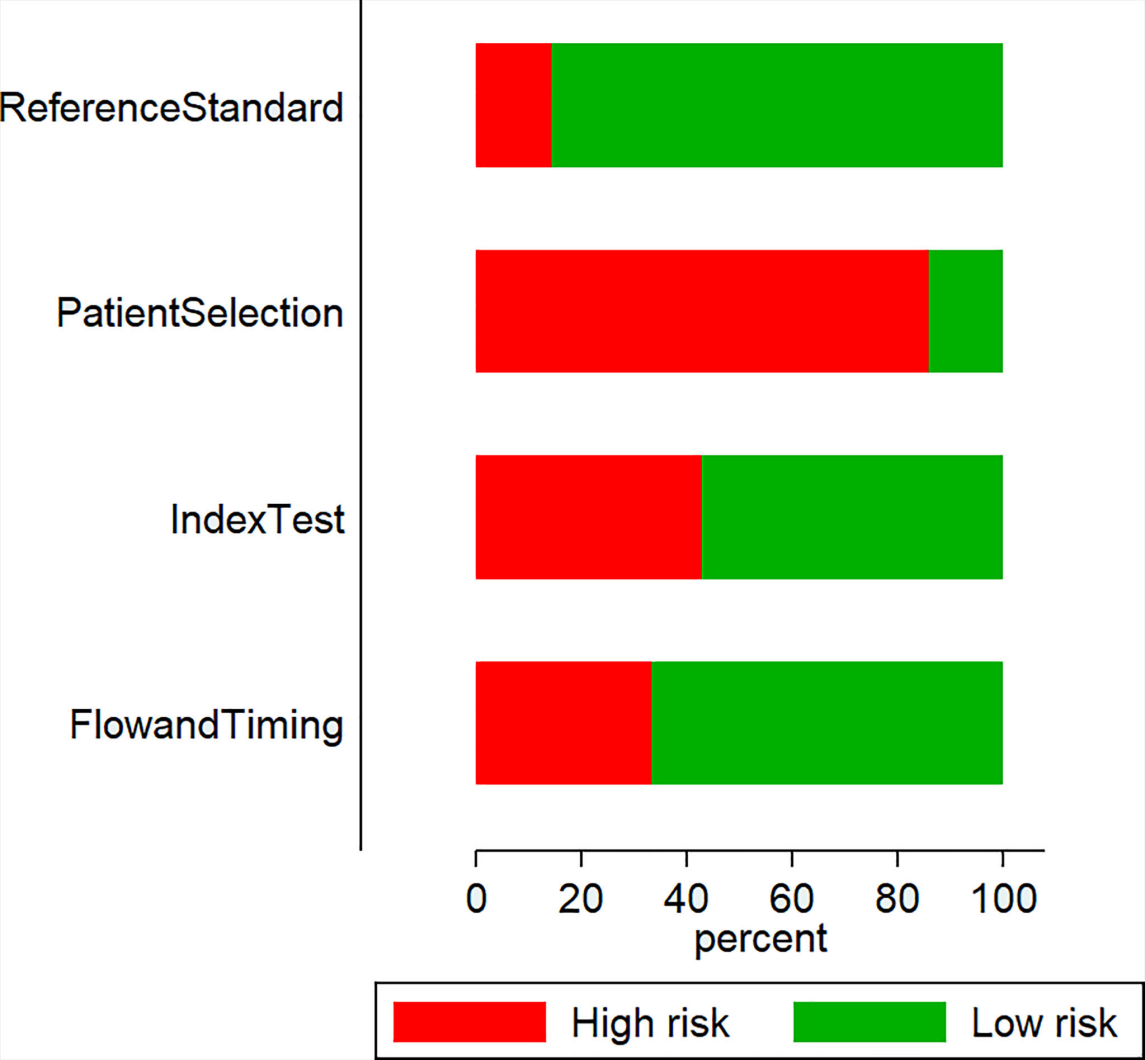
Quality assessment among the included studies using QUADAS-2 tool (n = 21).

We found that most studies (18 out of 21) presented high patient-selection risks: nine studies had high index test execution and interpretation bias risks; three studies had high reference standards bias risks; and seven had high patient flow and interval between index test and reference standard bias risks (**Figure 2**).

### Diagnostic Accuracy of Contrast-Enhanced MRI for Hepatocellular Carcinoma

We found 21 studies (10, 18–37) that had reported the accuracy of contrast-enhanced MRI for HCC diagnosis. However, we performed separate accuracy analyses based on the contrast agent and guidelines used by the different studies.

#### Accuracy of Gadoxetic Acid-Enhanced MRI

**Figure 3:**
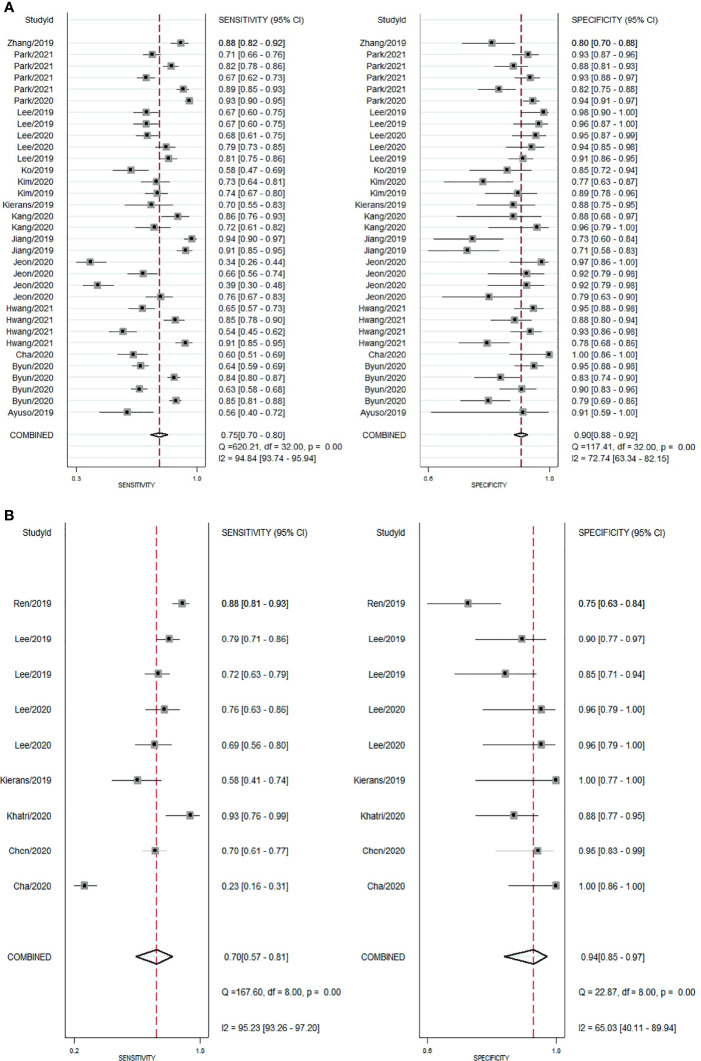
Forest plot showing pooled sensitivity and specificity for contrast-enhanced MRI. **(A)** gadoxetic acid-enhanced MRI. **(B)** ECA-MRI. ECA, extracellular contrast agent.

**Figure 4:**
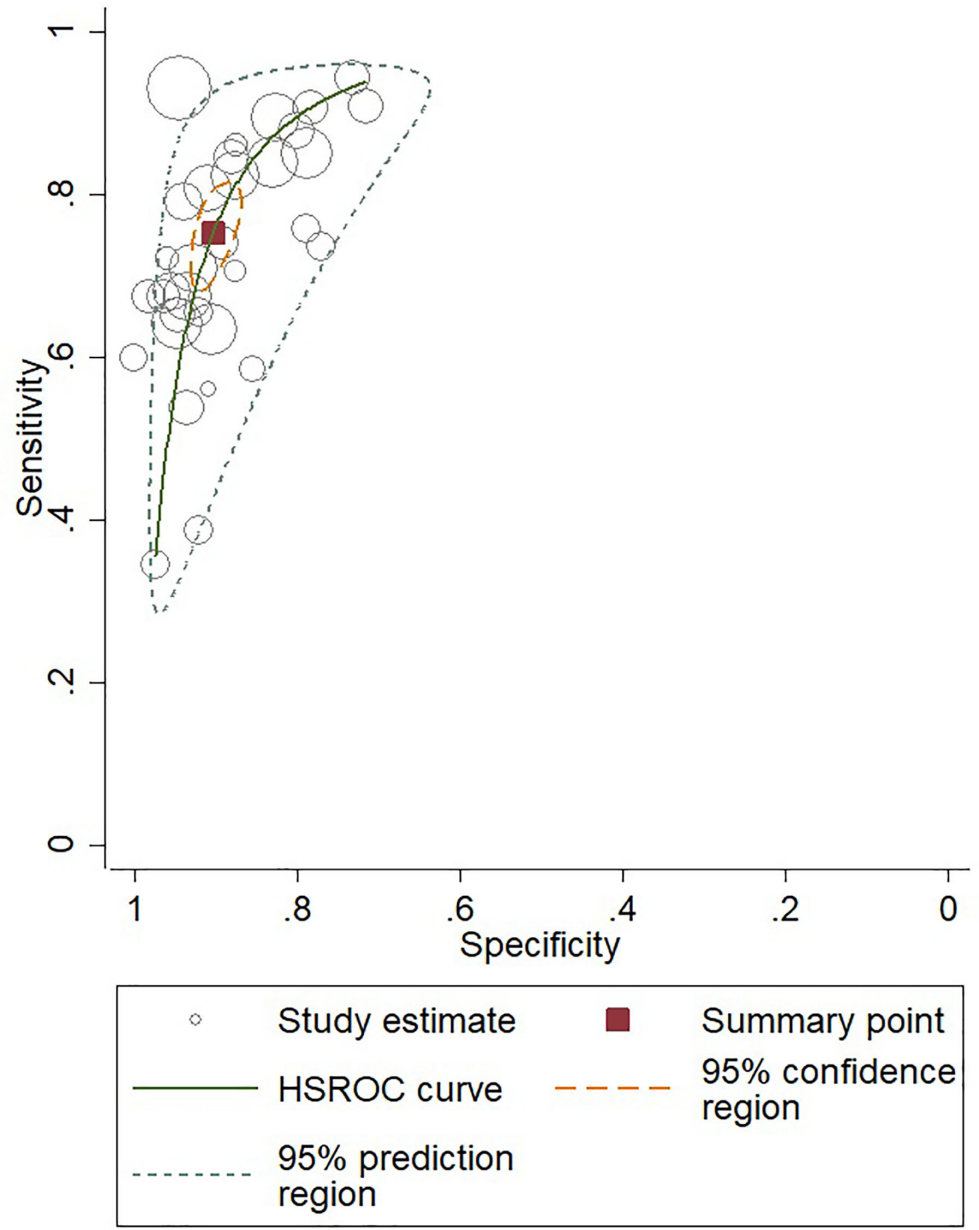
SROC curve for gadoxetic acid-enhanced MRI for HCC diagnosis. SROC, summary receiver operator characteristic curve; HCC, hepatocellular carcinoma.

**Figure 5:**
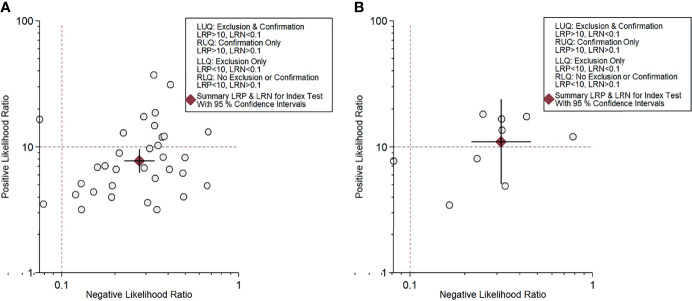
Likelihood scattergram for contrast-enhanced MRI. **(A)** Gadoxetic acid-enhanced MRI. **(B)** ECA-MRI. ECA, extracellular contrast agent.

**Figure 6:**
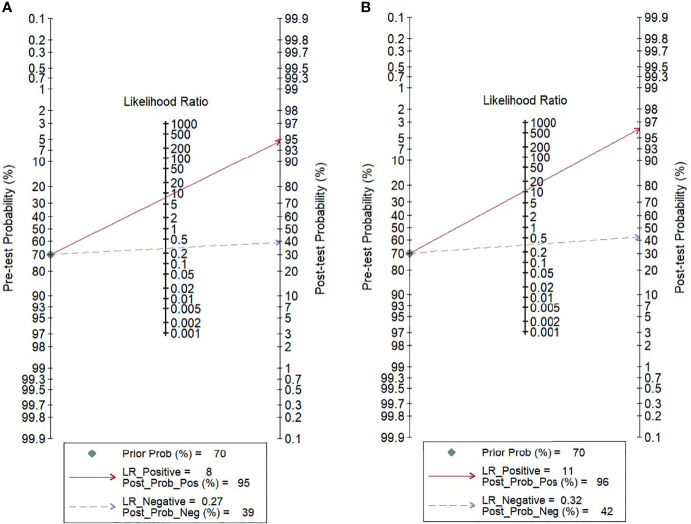
Fagan’s nomogram evaluating the overall value of contrast-enhanced MRI for the diagnosis of HCC. **(A)** Gadoxetic acid-enhanced MRI. **(B)** ECA-MRI. HCC, hepatocellular carcinoma; ECA, extracellular contrast agent.

**Figure 7:**
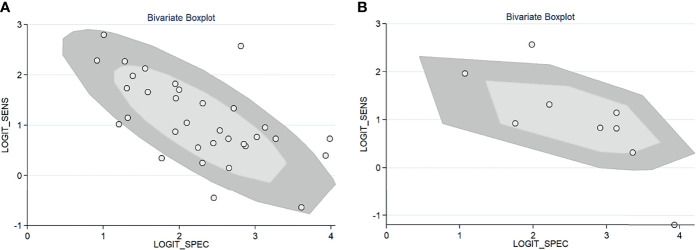
Bivariate boxplot of the sensitivity and specificity in the included studies. **(A)** Gadoxetic acid-enhanced MRI. **(B)** ECA-MRI. ECA, extracellular contrast agent.

For gadoxetic acid-enhanced MRI, the pooled sensitivity for diagnosing HCC was 75% (95% CI, 70%–80%) and the pooled specificity was 90% (95% CI, 88%–92%) (**Figures 3A** and **4**). The DOR was 28 (95% CI, 22–36). The +LR was 7 (95% CI, 6–29), and the −LR was 0.27 (0.22–0.33). Our LR scattergram (**Figure 5A**) shows that the +LR and −LR are in the right lower quadrant, indicating that the gadoxetic acid-enhanced contrast MRI cannot be used for HCC confirmation or exclusion. Fagan’s nomogram (**Figure 6A**) shows a moderate clinical utility of gadoxetic acid-enhanced contrast MRI for HCC diagnosis (Positive = 95%; Negative = 39%) that is significantly different from the pretest probability (70%). We found a significant between-study variability with a chi-square *p*-value of <0.001 and an *I* ^2^ value >75%. **Figure 7A** further confirms these findings in the relevant bivariate boxplot.

**Figure 8:**
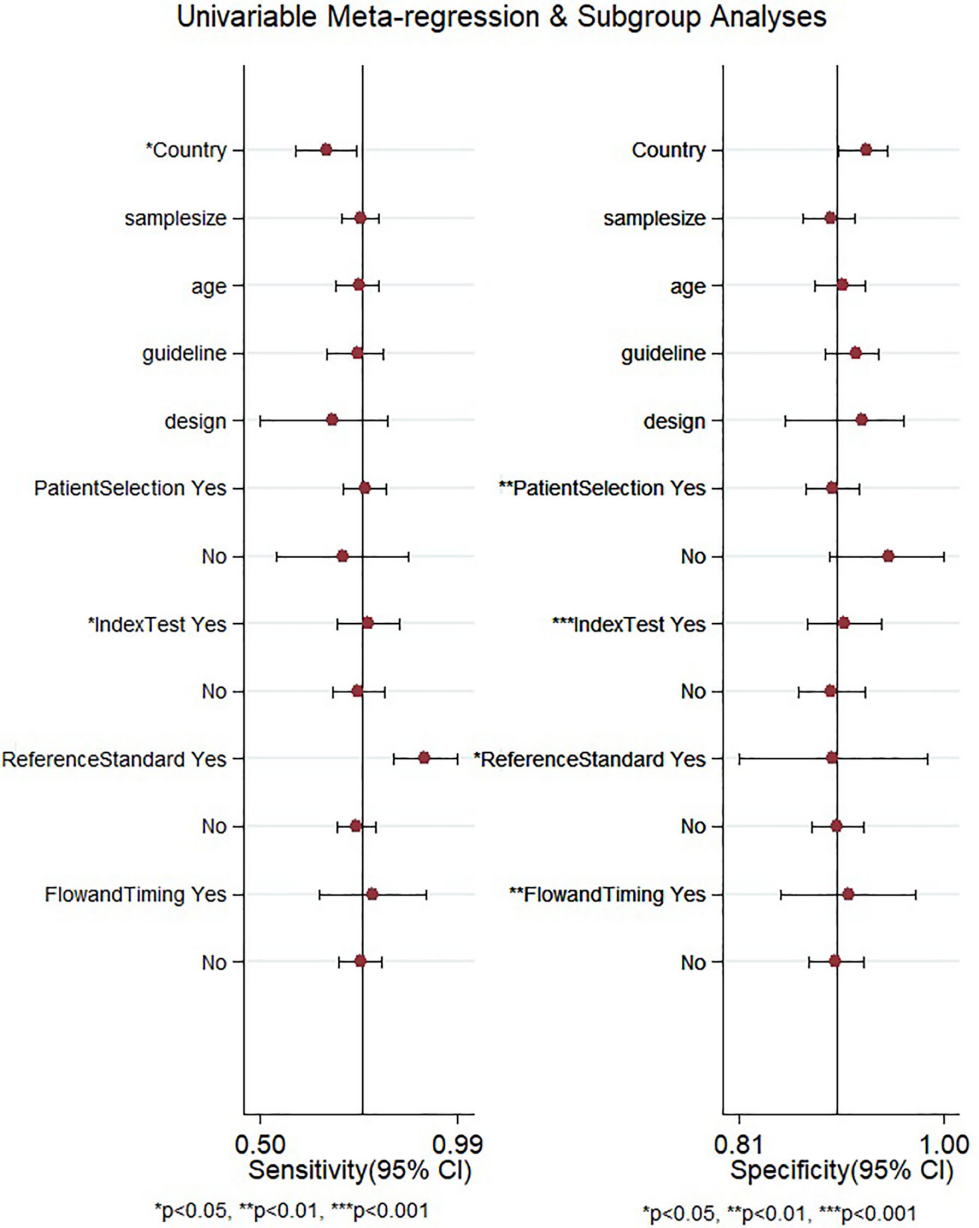
Meta-regression to explore the source of heterogeneity among the studies reporting gadoxetic acid-enhanced MRI for diagnosing HCC. HCC, hepatocellular carcinoma.

**Figure 9:**
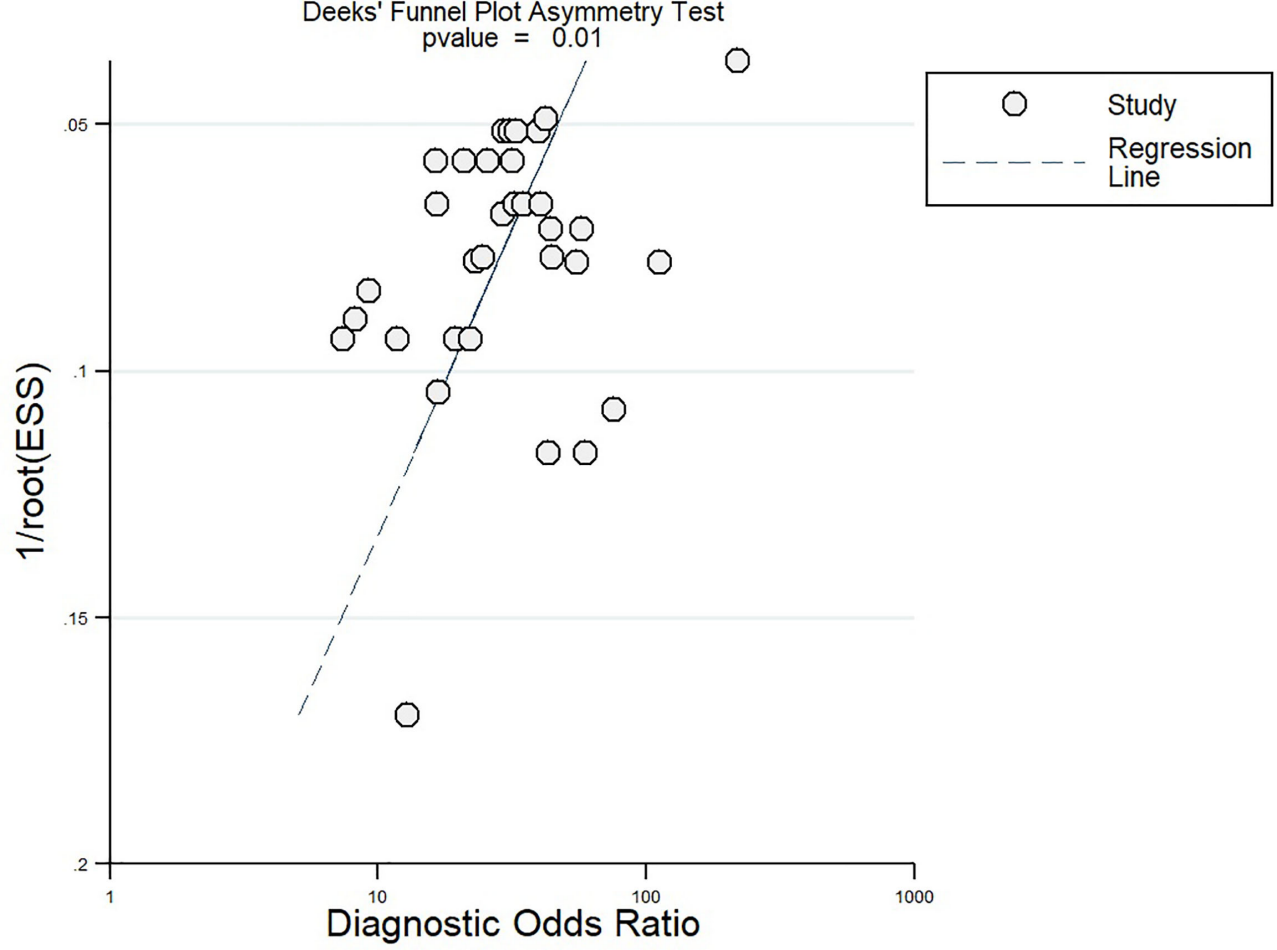
Funnel plot for publication bias.

After the meta-regression to explore the source of heterogeneity using potential covariates (**Figure 8**), our results indicated that the country (*p* < 0.05) may be a potential source of heterogeneity in the sensitivity model, while all the four quality assessment parameters (*p* < 0.001) were significant heterogeneity sources in the specificity model. The heterogeneity sources in the joint model included the country, sample size, and reference standard execution and interpretation (*p* < 0.001). Deeks’ test for publication bias showed a significant *p*-value (*p* = 0.01) indicating the presence of publication bias. This was further confirmed by the asymmetrically shaped funnel plot (**Figure 9**).

#### Accuracy of Extracellular Contrast Agent–MRI

The pooled sensitivity and specificity of ECA-MRI for diagnosing HCC were 70% (95% CI, 57%–81%) and 94% (95% CI, 85%–97%), respectively (**Figure 3B**). The LR scattergram (**Figure 5B**) shows that the +LR and −LR are in the right upper quadrant, indicating that the ECA-MRI can be used for HCC confirmation, but not for HCC exclusion. Fagan’s nomogram (**Figure 6B**) shows good clinical utility of gadoxetic acid-enhanced contrast MRI for HCC diagnosis (Positive = 96%; Negative = 42%) that is significantly different from the pretest probability (70%). We found a significant between-study variability with a chi-square *p*-value <0.001 and an *I* ^2^ >75%. The bivariate boxplot further confirmed these findings (**Figure 7B**). We could not perform a meta-regression (for source of heterogeneity) or a Deeks’ test (for publication bias) because we had less than the requisite 10 studies to perform the analyses.

#### Subgroup Analysis

We performed subgroup analyses based on the guidelines used for HCC diagnosis. The pooled sensitivity and specificity of contrast-enhanced MRI using AASLD for diagnosing HCC were 82% (95% CI, 71%–81%) and 81% (95% CI, 75%–86%), respectively; the same pooled values using EASL were 67% (95% CI, 51%–80%) and 91% (95% CI, 84%–95%), respectively; those using KLCSG-NCC were 78% (95% CI, 72%–82%) and 90% (95% CI, 86%–93%), respectively; and those using LIRADS were 73% (95% CI, 65%–80%) and 92% (95% CI, 89%–95%).

The sensitivity analysis did not reveal any significant small-study effects affecting the pooled sensitivity or specificity of any reported outcomes.

## Discussion

Contrast-enhanced MRI has been a reliable tool for the identification of HCC, irrespective of the risk of malignancy of the patients. However, the technique has been most commonly applied in cases with comorbid conditions such as liver cirrhosis or chronic hepatitis infections. The technique has several advantages for diagnosing HCC including a short execution time, its ease of performance, and its reduced healthcare cost when compared with the cost of diagnostic invasive procedures. However, the evidence synthesizing the accuracy of this technique is lacking, especially depending on the type of contrast agents and current guidelines used. Thus, we designed this review to assess the diagnostic accuracy of contrast-enhanced MRI for HCC.

From our literature review, we found 21 studies reporting the diagnostic utility of contrast-enhanced MRI for patients with HCC. Most studies were retrospective in nature and had low bias risks in most domains (except for patient selection, which presented high bias). Most studies originated in Eastern countries. HBV infection is more frequent in the Eastern parts of the world, where the hepatitis B vaccination rates have remained low and the incidence of HBV-related cirrhosis remains high.

We found that gadoxetic acid-enhanced MRI had a better sensitivity than ECA-MRI (75% *vs.* 70%), but ECA-MRI had a better specificity than gadoxetic acid-enhanced MRI (94% *vs*. 90%). However, the difference was modest, indicating that contrast-enhanced MRI has good accuracy for diagnosing HCC irrespective of the type of the contrast agent used. Other accuracy variables also showed high HCC diagnostic accuracy values for both types of contrast-enhanced MRI techniques. The clinical utility of both contrast-enhanced MRI techniques was high as evidenced by the significant rise in the posttest probability compared with the pretest probability in the Fagan’s nomogram. However, we found that ECA-MRI occupied the right upper quadrant in the LR scattergram, while gadoxetic acid-enhanced MRI occupied the right lower quadrant. This indicates that ECA-MRI can be used for HCC confirmation, but gadoxetic acid-enhanced MRI cannot be used for either confirmation or exclusion.

To the best of our knowledge, no review for ECA-MRI in HCC diagnosis exists; however, Li et al. conducted a meta-analysis on the diagnostic accuracy of gadoxetic acid-enhanced MRI for HCC, and their results showed slightly better values for sensitivity (85%) and specificity (94%) (38) than did ours. This difference in findings can be attributed to the fact that the previous review included only eight studies and to the difference in diagnostic guidelines used. Hence, we performed a subgroup analysis based on the type of diagnostic guidelines used and found that the studies based in AASLD had better sensitivity (82%), and the studies based on LIRADS had better specificity (92%) than had the others. Other reviews have also synthesized the findings from LIRADS guidelines and reported similar sensitivities and specificities to those in our study (39, 40).

Based on our findings, both types of contrast-enhanced MRI technique using any of the current set of guidelines can be used for HCC surveillance and diagnostic purposes. However, ECA-MRI with LIRADS guidelines is the only combination that can be used to confirm HCC due to the higher specificity it afford as compared with that by others. Other studies should update and compare the diagnostic performance of contrast-enhanced MRI with that of similar imaging techniques. Moreover, large-scale longitudinal studies are required to confirm the diagnostic accuracy of contrast-enhanced MRI, as most existing studies are retrospective in nature.

Our findings have several clinical implications because identifying patients with HCC early can improve the outcomes of medical interventions. In addition, based on our results, radiologists and clinicians should be able to apply the right diagnostic technique to obtain an accurate disease staging to select the appropriate therapeutic management. Different mathematical models for imaging techniques have also demonstrated that MRI offers the most cost-effective diagnostic technique for HCC (16).

Our results need to be interpreted with caution due to the differing qualities and methods used among the studies analyzed, which may have influenced our final summary findings. We found significant between-study variability (significant chi-square test and *I* ^2^ statistic). This heterogeneity can be attributed to the various ethnicities of the study participants and their variable risk factors and severities. Deeks’ test and the funnel plot showed the absence of publication bias among the studies reporting the diagnostic accuracy of contrast-enhanced MRI.

Our review is the first meta-analysis assessing the accuracy of both types of contrast-enhanced MRI and all the current guidelines for HCC diagnosis. Our analysis involved many studies with numerous participants (21 studies with more than 5,000 patients). In spite of these strengths, our review also had some limitations. First, we found a significant between-study variability in our analysis, which may have affected our interpretation of the pooled findings. Second, the diagnostic accuracy of contrast-enhanced MRIs may be affected by factors we could not consider such as the ethnicity of the participants, the timing of the assessment, and the associated risk factors. Third, we found a significant publication bias that may affect the credibility of our results. Finally, we did not analyze molecular features and histopathologic data that could determine specific imaging characteristics for different types of MRI technique (with or without hepato-specific contrast agents); however, we are aware that these data might explain the different results of previously published meta-analyses.

Despite these limitations, our findings provide valuable information for HCC diagnosis and have important clinical practice implications for patients with high risk of liver malignancy. Future studies should explore whether the contrast media used for MRI can help classify patients into intermediate-stage subclasses that have been shown to efficiently and reliably predict outcomes in untreated patients with HCC (41). Large-scale specific longitudinal studies are required to establish the best contrast-enhanced MRI method for evaluating patients admitted with liver cirrhosis, chronic hepatitis, or other high-risk chronic liver diseases.

## Data Availability Statement

Publicly available datasets were analyzed in this study. These data can be found in the databases of MEDLINE, EMBASE, Cochrane Library, Web of Science, SCOPUS, ScienceDirect, and Google Scholar from inception since January 2021.

## Author Contributions

CZ and HD conceived and designed the study. JS, QH, WS, and PW were involved in the literature search and data collection. QT, JRZ, SoX, and JJZ analyzed the data. CZ, HD and ShX wrote the paper. ShX reviewed and edited the manuscript. All authors contributed to the article and approved the submitted version.

## Conflict of Interest

The authors declare that the research was conducted in the absence of any commercial or financial relationships that could be construed as a potential conflict of interest.

## Publisher’s Note

All claims expressed in this article are solely those of the authors and do not necessarily represent those of their affiliated organizations, or those of the publisher, the editors and the reviewers. Any product that may be evaluated in this article, or claim that may be made by its manufacturer, is not guaranteed or endorsed by the publisher.
